# Bathing, Its Agency in Therapeutics

**Published:** 1887-07

**Authors:** B. M. Lawrence


### BATHING, ITS AGENCY IN THERAPEUTICS.

By B. M. Lawrence, M. D.

The body is made of cells; they may be regarded as the material, the brick or stone, out of which this human "temple wherein a God may dwell," is constructed. This tenement in which the soul lives for a few short years, has to undergo continual repairs, we sleep so that the worn-out cells can be taken up and replaced by new ones, which are continually being manufactured out of the food that we eat and of the air or oxygen which we breathe, and water is the vehicle which conveys the new material needed for repairs, and which removes the old, wornout tissues from the system. Water thus becomes in the body as it is in all nature, the great agent of purification, and since all disease is caused by, or in some way connected with impurity, the uses or *rationale* of water as a remedial agent, can be readily understood.

The body is not only made of cells, but these cells are all so arranged as to form tubes. Even the bones and the hairs are composed entirely of tubes, in which there is a continual circulation, a constant flow of fluids, of which water is the principal part. The skin contains many millions of these tubes, and in a full grown man their united length, it has been carefully estimated, would amount to not less than twenty-eight miles. Through these tubes not less than one or two pounds of matter pass daily from the body in the form of insensible perspiration. Bathing, oiling and rubbing the skin keep these little tubes open and active, and permits impurities to escape, which, if retained, would render the blood dark colored, and charge it with catarrhal phlegm which would tend to retard the circulation, cause congestion, and lay the foundation for consumption and many other forms of disease.

Not only should we regularly bathe the surface of our bodies for the purpose of cleanliness, as above indicated, but the entire tubing of the whole body should be abundantly supplied with this natural agent of purification. Pure, ripe, juicy fruits furnish the best, most wholesome and agreeable supply of water for the system, and there are few people who use one-quarter as much fruit, as a state of perfect health would indicate or demand, but this fruit should be taken at, or form the principle part of our meals and never be eaten between meals, especially so, if more than two regular meals are taken daily. Hot water drinking which has amounted almost to a mania, in many places during the past few years, was nothing more nor less than internal bathing. Suppose the system is filled with some form of impurity, causing congestion and disease, the patient drinks one, two or even three quarts of water daily, as many do who visit the springs and watering places. The same quantity of water must pass out of the system through the skin, kidneys or some other emunctories of the body, and in no case does it pass out as pure water, but becomes loaded with effete matter, which it takes up and expels.

There are many people who go to great expense to visit these resorts, who would have been equally as much benefited, if not far more so, had they remained at home and practiced daily bathing of their bodies, both internally and externally, by the use of this great natural therapeutic combined with sun-bathing, outdoor exercise, rest, recreation with abundance of oxygen in the form of pure, fresh air, by day and night. The following rules may be regarded as applicable to all cases, but more especially so to invalids and all who are not possessed of robust health and vital energy :

1 Never bathe just before or immediately after eating ; a full bath should not be taken less than an hour before, and two or three hours after partaking of a hearty meal

2. Before taking a cold bath, always see that the feet are made warm by hot water, by the fire or by exercise, but do not become fatigued before bathing, which might prevent a reaction.

3. Avoid drinking cold water or becoming chilled before taking any cold bath. While taking a hot bath, cold water may be supped freely, but in most cases, hot water is preferable, especially if the object of the bath is to produce perspiration.

4. After bathing, the whole body should be rubbed with the hand, using a very little oil, three parts olive and one part each of cajeput, sassafras and wintergreen, flavored with oil of cedar or to suit the fancy, makes a good mixture.

5. Care should be taken not to allow the feet to become cold, or to become chilled after bathing ; patients, if not able to exercise after bathing, should be warmly covered up in bed for an hour or two.

6. Persons who are naturally delicate, and all invalids who are feeble and debilitated, should carefully avoid all very cold, very hot, very long or unpleasant baths, especially severe shocks of shower or douche baths.

7. Local baths taken by sitting in a bath tub or common wash tub tipped over on one edge, with only a pailful of warm, cool or cold water in it, with the feet in a pail filled with hot water, taken as often as once or twice a day, using at the same time a weak solution of the sulphite of soda, as an injection, will cure one of woman's worst complaints when all the doctors drugs are unavailing.

8. A wet bandage made out of an old sheet or two or three yards of cotton flannel, one-half wet in hot, cool or cold water and wrapped about the hips at bedtime, surrounded with the dry part to protect the bedding, has cured the worst cases of seminal and female weakness, of spermatorrhœa and leucorrhœa, after a few weeks or months trial in cases where all other remedies failed to benefit.

9. Whatever the form of bath when taken regularly, it should be omitted once or twice a week, and the temperature of the bath should be carefully regulated ; where a tonic effect is desired, the less heat the better, always avoiding any very disagreeable sensations.

10. Particular attention should be paid to the temperature of the bathing room, and also to the ventilation. For invalids, the temperature should be about seventy-five degrees. The sun bath is one of the best tonics in the entire list of nature's remedies, and some future day, will doubtless be so regarded, but of this agency, also of sea or surf bathing, more will be found in future chapters of HALL'S JOURNAL.

